# The Role of *BMP7* in the Proliferation of Hu Sheep Dermal Papilla Cells Is Influenced by DNA Methylation

**DOI:** 10.3390/ani14111699

**Published:** 2024-06-05

**Authors:** Xiaoyang Lv, Mingliang He, Shanhe Wang, Wenxin Zheng, Hanlin Zhou, Joram M. Mwacharo, Wei Sun

**Affiliations:** 1 Joint International Research Laboratory of Agriculture and Agri-Product Safety of Ministry of Education of China, Yangzhou University, Yangzhou 225009, China; dx120170085@yzu.edu.cn; 2 College of Animal Science and Technology, Yangzhou University, Yangzhou 225009, China; 3 International Joint Research Laboratory in Universities of Jiangsu Province of China for Domestic Animal Germplasm Resources and Genetic Improvement, Yangzhou University, Yangzhou 225009, China; 4 Xinjiang Academy of Animal Sciences, Urumqi 830011, China; 5 Zhanjiang Experimental Station, Chinese Academy of Tropical Agricultural Sciences, Zhanjiang 524000, China; 6 International Centre for Agricultural Research in the Dry Areas, Addis Ababa 999047, Ethiopia

**Keywords:** Hu sheep, lamb skin, BMP7 gene, methylation, dermal papilla cell

## Abstract

**Simple Summary:**

Dermal papilla cells (DPCs) play a key role in the growth and development of hair follicles, and their number can regulate the shape and size of hair. Previous studies found that the *BMP7* gene is differentially expressed in Hu sheep lamb skin of different pattern types and plays a key role in the proliferation of Hu sheep DPCs. However, the molecular mechanism of the differential expression of the *BMP7* gene remains unclear. Here, we aim to investigate the cause of the *BMP7* gene differentially expressed in Hu sheep lamb skin of different pattern types. Our findings demonstrate that DNA methylation modification can affect the expression of the *BMP7* gene and can regulate the proliferation and cell cycle of Hu sheep DPCs.

**Abstract:**

Previous studies have shown that the *BMP7* gene is differentially expressed in Hu sheep lamb skin of different pattern types, and its expression level is significantly correlated with hair follicle indices of different pattern types, but the molecular mechanism of the differential expression of the *BMP7* gene remains unclear. This study investigated the effect of DNA methylation on the transcriptional expression of *BMP7*. Firstly, we found that the mRNA expression of the BMP7 gene and the activity of the core promoter of the BMP7 gene were upregulated after 5-Aza-Deoxycytidine-induced demethylation treatment using qRT-PCR and double luciferase reporter assay. Then, we found that the proliferation of Hu sheep DPCs in vitro was promoted after 5-Aza-Deoxycytidine-induced demethylation treatment through qRT-PCR, CCK-8, and EdU assay, and that the overexpression of DNMT1 in DPCs induced the opposite effect. In addition, the results of the cell cycle assay reveal that the percentage of cells in the S phase was increased after 5-Aza-Deoxycytidine-induced demethylation treatment, and that the percentage of cells in the S phase was decreased after overexpression of DNMT1 in DPCs. This study indicated that the differential expression of the *BMP7* gene in different patterns of Hu sheep lamb skin may be regulated by DNA methylation modification. In addition, DNA methylation can regulate the proliferation and cell cycle of DPCs in Hu sheep.

## 1. Introduction

Hu sheep is a local breed of sheep in China, with good characteristics such as high fertility, high lambing rate, and perennial estrus. In addition, the white lamb skin produced by Hu sheep has the characteristics of a wavy pattern and being world-renowned [1]. The wavy pattern of Hu sheep lamb skin is mainly formed by the bending of wool, which determines the quality of Hu sheep lamb skin to a certain extent.

Wool formation is closely related to the growth and development of hair follicles. Hair follicles are small accessory organs of the skin, containing many types of cells such as dermal papilla cells, inner root sheath, and outer root sheath [2,3,4]. It is generally believed that the bending of wool is mainly caused by the asymmetric proliferation of various cells in the hair follicle, especially the asymmetric growth of cells on both sides of the dermal papilla [5]. DPCs are the dermal part of the hair follicle, located at the base of the hair follicle and surrounded by hair matrix cells. As the signal center, the DPCs send signals to the hair matrix cells to affect the differentiation of the hair matrix cells and the growth and development of hair [6,7]. Chi et al. found that the number of DPCs in the hair follicle is directly related to the shape of the hair in a mouse model [8]. They first found that there were significant differences in the number of DPCs in different hair types, that the number of DPCs contained in “acicular” hair follicles is significantly lower than that in “shaped” hair follicles, and that the shape of the hair also changed with the number of DPCs throughout successive hair cycles. In addition, the straight hair of a mouse will change to “shaped hair” by using conditional knockout methods in mice to specifically reduce the number of DPCs [8]. Rompolas et al. found that hair follicles could not enter the growth phase and the shape of the hair changed after specifically eliminating DPCs using lasers [9]. These studies suggest that the number of DPCs in the hair follicle affects the hair’s curved phenotype.

With the deepening of the research on the formation of hair curvature, the gene and molecular regulatory mechanisms affecting hair follicle growth and development and hair shape have been found. Studies have shown that the expression and secretion of various growth factors and intercellular signaling molecules in DPCs can affect the formation of a hair follicle symmetry/asymmetry axis, including Wnt, TGF-β/BMP, FGFs, and other factors with regulatory roles [10]. In addition, the regulatory mechanism of hair curvature is closely related to hair follicle growth and development pathways, such as Wnt/β-Catenin and TGF-β pathways [3,11,12]. The bone morphogenetic protein (BMP) is a functional protein belonging to the TGF-β superfamily, which plays an important role in hair follicle growth and development [13]. Previous studies have shown that *BMP7* is a differentially expressed gene in different pattern types of Hu sheep lamb skin, and its expression level is significantly correlated with hair follicle indicators, such as the number and diameter of primary and secondary hair follicles [14]. In addition, *BMP7* can promote the proliferation of Hu sheep dermal papillae cells and inhibit the TGF-β/Smad signaling pathway [15]. These studies suggest that *BMP7* may be involved in the growth and development of Hu sheep hair follicles by influencing the proliferation of Hu sheep DPCs, and thus affect wool bending.

DNA methylation modification is one of the important ways to regulate gene expression, and it is involved in many metabolic processes during body growth and development [16]. DNA methylation also plays an important role in hair growth and hair follicle development. Zhu et al. found that the methylation of the lncRNA-H19 promoter region is likely to participate in the transcriptional inhibition of secondary hair follicles in Liaoning cashmere goats, thus regulating hair follicle growth and development [17]. Tian et al. found that the methylation level and expression level of the Wnt gene were different in different stages of hair follicle growth and development by using MeDIP-seq to obtain the genome-wide methylation map of hair follicle development of Merino sheep [18]. Bai et al. found that the methylation frequency of the fifth CpG site on the CpG island in the promoter region of Wnt10b was lower in the growth stage than the regression stage and, in the resting stage in the secondary hair follicles of Angolan rabbits, the methylation level was negatively correlated with the expression of Wnt10b, which played a role in transcriptional inhibition [19]. However, there are relatively few studies on DNA methylation modification during the growth and development of Hu sheep hair follicles.

This study aimed to investigate the regulatory mechanism of the *BMP7* gene expression and the regulation of DNA methylation modification on DPC proliferation, further understanding the regulatory mechanism of the *BMP7* gene on DPC proliferation and its role in hair follicle growth and development.

## 2. Methods

### 2.1. Ethics Statement and Animals

The Animal Ethics Committee of Yangzhou University approved the experimental protocols (Approval number: No. 202103279). The experimental animals were healthy 3-day-old Hu sheep lambs with good growth conditions from a breeding sheep farm in Suzhou, Jiangsu Province. We collected three skin tissues from the back of Hu sheep lambs for cell isolation.

### 2.2. Cell Isolation, Culture, and Transfection

Hu sheep DPCs were isolated from the skin of Hu sheep lamb. First, the skin tissue taken from the Hu sheep scapular was cut into small pieces; then, the single hair follicle was isolated with tweezers, and its ends were destroyed. Finally, the incomplete hair follicle ends were transplanted into the medium to obtain the DPCs. The medium of DPCs was DMEM-F12 (Sigma-Aldrich, St. Louis, MO, USA) added with 10% fetal bovine serum and 1% penicillin–streptomycin–amphotericin. The growth environment of DPCs was 37 °C and 5% CO_2_. jetPRIME Transfection Reagent (Polyplus, Illkirch, France) was used for all cell transfection and transfection protocol was performed strictly according to the instructions.

### 2.3. Total RNA Extraction, cDNA Synthesis, and qRT-PCR

Total RNA was extracted from DPCs using Trizol (Takara, Dalian, China) and strictly following the instructions. The first strand of cDNA was synthesized using a Hiscript QRT Supermax (Vazyme, Nanjing, China) kit strictly following the instructions. The mRNA expression levels of genes were detected using a ChamQ SYBR qPCR Master Mix (Vazyme, Nanjing, China) kit strictly following the instructions. The sheep GAPDH gene was used as the housekeeping gene. A real-time fluorescence quantitative instrument (Bio-Rad, Hercules, CA, USA) was used to detect the mRNA expression level of related genes. In this experiment, each sample was repeat-tested three times. The relative expression of genes was calculated according to the 2^−ΔΔCT^ method [20].

### 2.4. Primers for qRT-PCR

All primers of related genes used for qRT-PCR assay in this study were designed using Premier Primer 5.0 software (Premier Biosoft International, Palo Alto, CA, USA). The primers of all related genes used for QRT-PCR assay in this study were synthesized by TsingKe (Nanjing, China). The correlated primer sequences are shown in Table 1.

### 2.5. Plasmids Construction

DNA was extracted from the skin tissue of Hu sheep, and the core promoter region of the *BMP7* gene was amplified using DNA as a template. First, the core promoter region fragment of the *BMP7* gene was amplified using a PrimeSTAR Max DNA Polymerase (Takara, Dalian, China). Then, the amplified product was detected by PCR by 1% agarose gel electrophoresis and was sequenced to confirm whether the amplified product was the target fragment. Finally, the restriction endonuclease NheI and HindIII were used for double digestion of the target fragment and pGL3-basic vector to construct the recombinant plasmid and the recombinant plasmid was finally extracted for sequence. The result of the DNA sequence was verified to be correct, indicating that the dual luciferase report vector of the core promoter region of the *BMP7* gene was successfully constructed.

Total RNA was extracted from the skin tissue of Hu sheep and reverse-transcribed into cDNA. The coding sequence (CDS) region of the *DNMT1* gene of Hu sheep was amplified using cDNA as a template. First, the CDS region fragment of the *BMP7* gene was amplified using a PrimeSTAR Max DNA Polymerase (Takara, Dalian, China). Then, the amplified product was detected by PCR by 1% agarose gel electrophoresis and was sequenced to confirm whether the amplified product was the target fragment. Finally, the restriction endonuclease EcoRI and NotI were used for double digestion of the target fragment and pcDNA 3.1 vectors to construct the recombinant plasmid, and the recombinant plasmid was finally extracted. The result of the CDS sequence was verified to be correct, indicating that the overexpression vector of the *DNMT1* gene was successfully constructed. The primers used for vector construction in this study were synthesized by TsingKe (Nanjing, China). The sequence of relevant primers is shown in Table 2.

### 2.6. 5-Aza-Deoxycytidine (5-Aza-dc)-Induced Demethylation

For the DNA demethylation treatment, DNA methyltransferase inhibitor 5-Aza-deoxycytidine(5-Aza-dc) (Monmouth Junction, NJ, USA) was used for cell culture, 5-Aza-dc was dissolved in dimethyl sulfoxide (Sigma, St. Louis, MO, USA). DPCs were cultured with 3 µg/mL 5-Aza-dc for 48 h for the subsequent experiments.

### 2.7. Dual-Luciferase Reporter Assay

First, the DPCs were inoculated on the 24-well cell culture plate. After the cell density reached 50%, the cells were transfected according to the instructions for the jetPRIME Transfection Reagent (polyplus, Illkirch, France). The experiment was divided into three groups: (1) the pGL3 basic group; (2) the PGL3-BMP7 promoter group; and (3) the PGL3-BMP7 promoter+5-Aza-dc group. After transfection for 24 h, the cells were collected and the relative luciferase activities of firefly luciferase and Renilla luciferase were detected according to the Dual-Luciferase Report kit (Vazyme, Nanjing, China), respectively. A Multimode micropore detection system (EnSpire, Perkin Elmer, Waltham, MA, USA) was used to measure the OD value of DPCs at 450 nm. The relative luciferase activities of firefly luciferase and Renilla luciferase were analyzed.

### 2.8. CCK-8 Assay

First, the DPCs were inoculated on the 12-well cell culture plate. After the cell density reached 50%, the cells were transfected according to the instructions for the jetPRIME Transfection Reagent. After transfection for 24 h, the DPCs were digested with trypsin and then added with an appropriate complete medium to prepare cell suspension. Then, the cell suspensions were inoculated into 96-well plates and cultured in a constant-temperature incubator. Finally, cell activity was detected at 12 h, 24 h, 36 h, and 48 h using a CCK-8 kit (Vazyme, Nanjing, China) according to the instructions for the CCK-8 assay. The steps were as follows: First, 10 μL CCK-8 detection reagent was mixed with a 90 μL DPC medium. Then, the original medium was sucked out, and the mixed medium was added to each well of the 96-well cell culture plate and cultured DPCs for 2 h before detection. A Multimode micropore detection system (EnSpire, Perkin Elmer, USA) was used to measure the OD value of DPCs at 450 nm.

### 2.9. EdU Assay

First, the DPCs were inoculated on the 24-well cell culture plate. After the cell density reached 50%, the cells were transfected according to the instructions for the jetPRIME Transfection Reagent. After transfection for 48 h, cell proliferation was detected using the EdU cell proliferation detection kit (RiboBio, Guangzhou, China) according to the instructions for the EdU assay. The steps are as follows: First, EdU assay A was mixed with DPC culture medium at a ratio of 1:1000. Then, the original medium was sucked out and the mixed medium was added to each well of the 24-well cell culture plate. Finally, the DPCs were fixed, permeability was conducted, and staining was performed after the cells were cultured for 2 h. An inverted fluorescence microscope (Nikon, Tokyo, Japan) was used for cell image acquisition.

### 2.10. Cell Cycle Assay

First, the DPCs were inoculated on the 6-well cell culture plate. After the cell density reached 50%, the cells were transfected according to the instructions for the jetPRIME Transfection Reagent. After transfection for 48 h, the cell cycle was detected using the cell cycle assay kit (Beyotime, Shanghai, China) according to the instructions. A FACSAria SORP flow cytometer (BD company, Franklin, NJ, USA) was used for cell cycle analysis.

### 2.11. Statistical Analysis

SPSS25.0 software (SPSS Inc., Chicago, IL, USA) was used for the *t*-test. * *p* < 0.05, ** *p* < 0.01, and *** *p* < 0.001 were considered as statistical significance. GraphPad Prism 8.0 (GraphPad Software Inc., San Diego, CA, USA) was used for data visualization. Three repeated tests were performed for each experiment, and the data were shown as means ± SEM (standard error of the mean).

## 3. Results

### 3.1. DNA Demethylation Upregulates the Expression and Transcriptional Activity of BMP7

**Figure 1:**
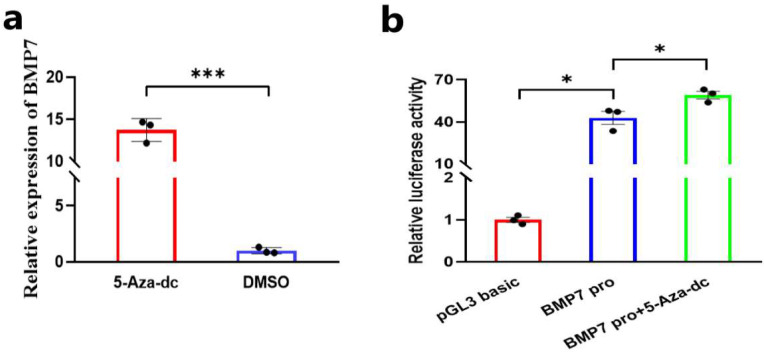
DNA demethylation upregulates the expression and transcriptional activity of *BMP7*. (**a**) The relative mRNA expression of *BMP7* after the DPCs were treated with 5-Aza-dc. (**b**) Analysis of the luciferase activity after the DPCs transfected with pGL3 basic, pGL3-BMP7-core promoter, and pGL3-BMP7-core promoter+5-Aza-dc, respectively. In all figures, data are shown as means ± SEM (standard error of the mean) (n = 3). The unpaired Student’s *t*-test was used for statistical significance (* *p* < 0.05; *** *p* < 0.001).

To explore the causes of the differential expression of the *BMP7* gene in different pattern types of Hu sheep lamb skin, DPCs were treated with 5-Aza-dc and then the mRNA expression level of the *BMP7* genes was detected. The results of qRT-PCR show that the mRNA expression level of the *BMP7* gene in DPCs treated with 5-Aza-dc was significantly upregulated (Figure 1a). In addition, we constructed a dual luciferase reporter vector containing the core promoter region of the *BMP7* gene based on our previous study [21]. Then, we detected the effect of 5-Aza-dc on the activity of the *BMP7* core promoter region by dual luciferase reporting assay. The result show that 5-Aza-dc treatment could upregulate the transcriptional activity of the *BMP7* gene core promoter region (Figure 1b). These results indicate that the differential expression of the *BMP7* gene in different pattern types of Hu sheep lamb skin may be regulated by DNA methylation modification.

### 3.2. DNA Demethylation Promotes the Proliferation of DPCs

**Figure 2:**
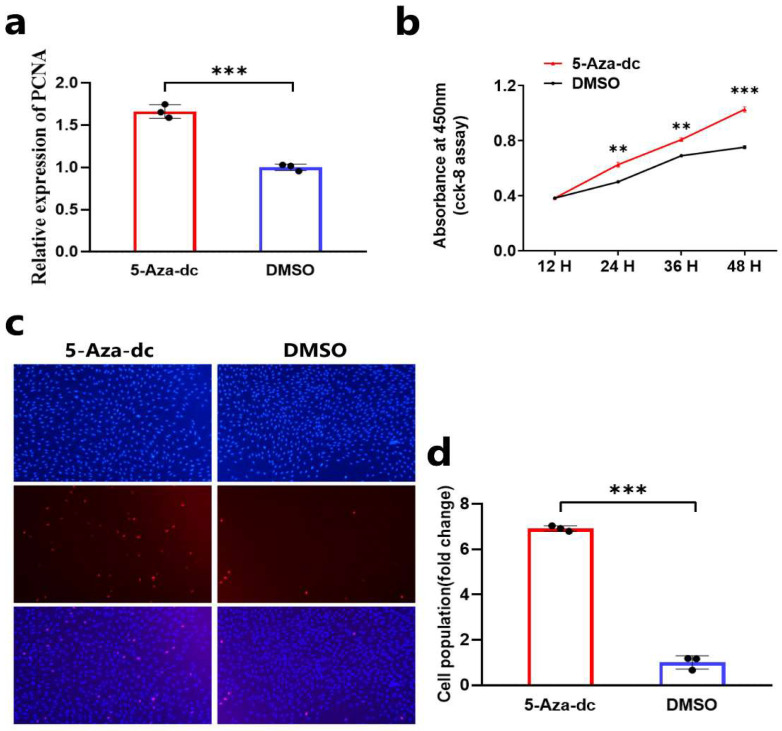
DNA demethylation promotes the proliferation of DPCs. (**a**) The relative mRNA expression of *PCNA* after the DPCs were treated with 5-Aza-dc. (**b**) CCK-8 assay after the DPCs were treated with 5-Aza-dc. (**c**,**d**) EdU assay after the DPCs were treated with 5-Aza-dc. In all figures, data are shown as means ± SEM (standard error of the mean) (n = 3). The unpaired Student’s *t*-test was used for statistical significance (** *p* < 0.01; *** *p* < 0.001).

To understand the effect of DNA demethylation on the proliferation of DPCs, we detected the proliferation of Hu sheep DPCs after 5-Aza-Deoxycytidine-induced demethylation treatment by qRT-PCR, CCK-8 assay, and EdU assay. The results of qRT-PCR show that the mRNA expression level of cell proliferation-related gene *PCNA* in DPCs after 5-Aza-dc treatment was upregulated (Figure 2a). CCK-8 assay results show that 5-Aza-dc treatment could promote the vitality of DPCs (Figure 2b). EdU assay results show that 5-Aza-dc treatment could promote the proliferation of Hu sheep DPCs in vitro (Figure 2c,d). These results suggest that DNA demethylation may promote the proliferation of Hu sheep DPCs in vitro by upregulating the expression of the *BMP7* gene.

### 3.3. DNA Methylation Inhibits the Proliferation of DPCs

**Figure 3:**
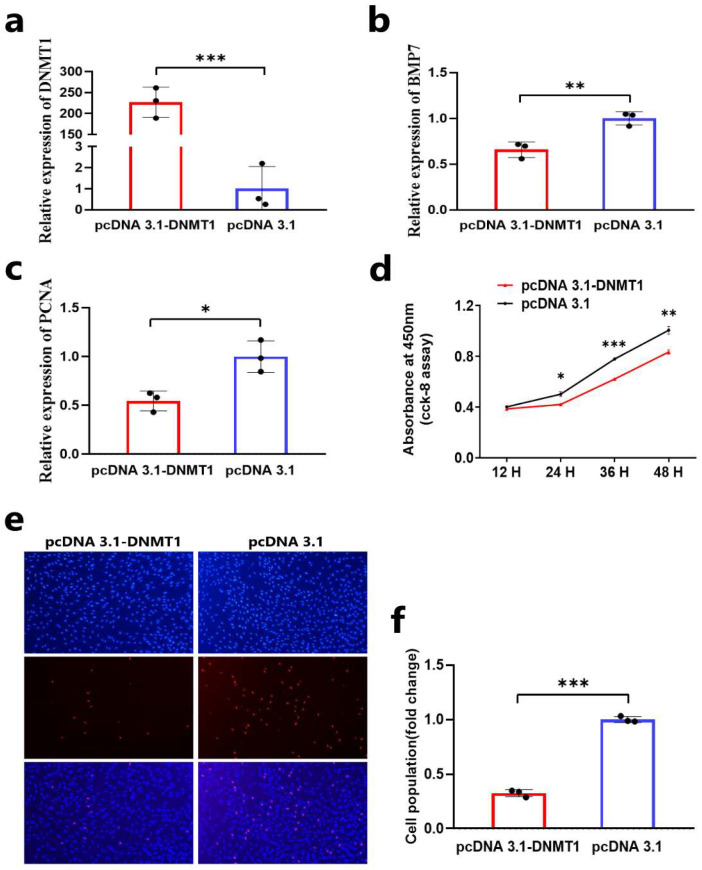
DNA methylation inhibits the proliferation of DPCs. (**a**) The relative mRNA expression of *DNMT1* after the DPCs transfected with pcDNA 3.1-DNMT1. (**b**) The relative mRNA expression of *BMP7* after the DPCs transfected with pcDNA 3.1-DNMT1. (**c**) The relative mRNA expression of *PCNA* after the DPCs transfected with pcDNA 3.1-DNMT1. (**d**) CCK-8 assay after the DPCs transfected with pcDNA 3.1-DNMT1. (**e**,**f**) EdU assay after the DPCs transfected with pcDNA 3.1-DNMT1. In all figures, data are shown as means ± SEM (standard error of the mean) (n = 3). The unpaired Student’s *t*-test was used for statistical significance (* *p* < 0.05; ** *p* < 0.01; *** *p* < 0.001).

We also detected the regulatory effect of DNA methylation on the process of proliferation of DPCs. First, we transfected the overexpression vector of *DNMT1* and found that the expression of the *DNMT1* gene was significantly upregulated in DPCs (Figure 3a). Then, we detected the expression of the *BMP7* gene and cell proliferation after overexpression of DNMT1 in Hu sheep DOCs by qRT-PCR, CCK-8 assay, and EdU assay. qRT-PCR showed that the mRNA expression level of the *BMP7* gene and cell proliferation-related gene *PCNA* were downregulated (Figure 3b,c). CCK-8 assay showed that *DNMT1* could inhibit the activity of DPCs (Figure 3d). EdU assay showed that *DNMT1* could inhibit the proliferation of DPCs in vitro (Figure 3e,f). These results suggest that DNA methylation may inhibit the proliferation of Hu sheep DPCs in vitro by downregulating the expression of the *BMP7* gene.

### 3.4. DNA Methylation Inhibits the Cell Cycle of DPCs

**Figure 4:**
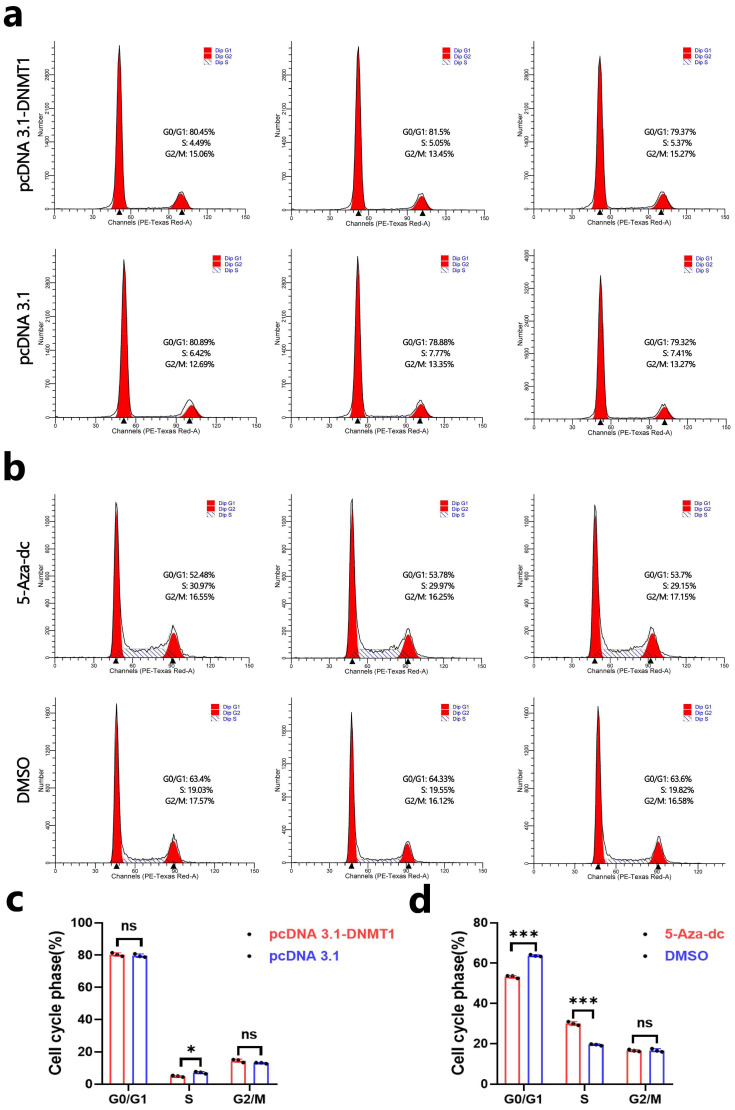
DNA methylation inhibits the cell cycle of DPCs. (**a**,**c**) The cell cycle was analyzed using a FACSAria SORP flow cytometer, and the G1, S, and G2 phases were counted after DPC treatment with pcDNA 3.1-DNMT1/pcDNA 3.1. (**b**,**d**) The cell cycle was analyzed using a FACSAria SORP flow cytometer, and the G1, S, and G2 phases were counted after DPC treatment with 5-Aza-dc/DMSO. In all figures, data are shown as means ± SEM (standard error of the mean) (n = 3). The unpaired Student’s *t*-test was used for statistical significance (^ns^ *p* > 0.05; * *p* < 0.05; *** *p* < 0.001).

In addition, we further detected the effect of DNA methylation modification on the cell cycle of DPCs. First, we found that the overexpression of DNMT1 could decrease the percentage of cells in the S phase (Figure 4a,c). Then, we also detected the effect of DNA demethylation treatment on the cell cycle of DPCs, and the results show that 5-Aza-Deoxycytidine-induced demethylation treatment could increase the percentage of cells in the S phase and decrease the percentage of cells in the G0/G1 phase (Figure 4b,d). These results suggest that DNA methylation can inhibit the cell cycle of Hu sheep DPCs in vitro by decreasing the percentage of cells in the S phase.

## 4. Discussion

The formation of the Hu sheep lamb skin pattern is closely related to wool bending, which is determined by hair follicle growth and development. As the “source” of wool growth, hair follicles regulate the cyclical changes in wool and affect the formation of wool bending. Hair follicles include hair papilla, hair matrix, inner root sheath, outer root sheath, and other components, which determine the growth and development of hair follicles and morphogenesis [22]. The growth and development of hair follicles depend on the interaction between epidermal and dermal cells of hair follicles. The DPCs are the source of hair follicle growth and differentiation, which determines the direction of hair follicle differentiation, the number of hair matrix cells, the size of hair shafts, and the elasticity and elongation of hair. Wool bending is opposite to the bending direction of hair follicle balls and the curvature of the hair follicle is strongly associated with the asymmetric growth of cells on both sides of the dermal papilla [23,24,25]. Meanwhile, Nissimov et al. proposed the multiple papillary center model (MPC), which refers to two or more growth-induced dermal papilla regions. Combined with the morphological characteristics of the dermal papilla isolated from a single hair follicle in the cross-section of hair, the model indicated that these dermal papilla could induce different growth rates of cortical cells in their adjacent regions. This explains the changes in hair growth, hair morphology, and hair curvature [3]. Therefore, we consider DPCs as the core of the study of wool bending and Hu sheep lamb pattern formation, and it is necessary to carry out further functional verification and molecular mechanism research on DPCs.

More and more studies have found that some genes are involved in hair-bending regulation. Many keratin and keratin-related proteins were found in the wool fibers of sheep and goats, which played an important role in wool fiber structure formation and were closely related to wool bending. Studies have shown that the mutation of SNP sites on *KRTAP20-2* and *KRTAP22-1* genes can affect wool bending [26,27]. *KRT25* and *KRT71* have also been found to play important roles in the formation of curly hair in horses and dogs, respectively [28,29]. Furthermore, Kang et al. found that *KRT71* is also one of the important regulatory genes involved in wool bending formation [30]. Besides the keratin family, the role of the BMP family in wool growth and development cannot be ignored. The BMP family protein is a secreted protein, one of the members of the TGFβ superfamily, which can regulate the proliferation, apoptosis, and differentiation of skin hair follicle cells, and affect the growth and development of hair follicles [13,31]. Studies have shown that the overexpression of *BMP2* could increase the expression of differentiation-related markers and decrease the expression of proliferation-related markers in hair follicle stem cells, thus promoting the differentiation process of hair follicle stem cells [32,33]. BMP can act as an inhibitor of the activation of hair follicle stem cells and has an inhibitory effect on the growth and development of hair follicles. Interestingly, when hair follicles are activated and start to grow, the expression levels of *BMP4* and *BMP6* will increase, which seems to be contrary to the theory that BMP signals are inhibitory signals; furthermore, the researchers found that this is a negative feedback loop [34,35,36]. Although there are no reports of BMP signaling on wool or hair bending, BMP signaling plays an important role in the growth and development of hair follicles, and it is also very likely to play a regulatory role in the formation of wool bending.

In a previous study, *BMP7* was found to be differentially expressed in different pattern types of Hu sheep lamb skin and to be able to promote the proliferation of Hu sheep DPCs in vitro [14,15]. This finding suggests that the *BMP7* gene may play a role in the growth and development of hair follicles by influencing the proliferation of DPCs. However, the exact regulatory mechanism of the *BMP7* gene remains unclear. To further understand the regulatory mechanism of the *BMP7* gene in the proliferation process of DPCs, we explored the factors influencing the differential expression of the *BMP7* gene in this study. In mammalian genomes, DNA methylation is defined as an epigenetic mechanism capable of regulating gene expression by recruiting proteins involved in gene suppression or inhibiting the binding of transcription factors to DNA [37]. Therefore, we aimed to explore the effect of DNA methylation modification on *BMP7* gene expression in this study, to understand the differential expression mechanism of the *BMP7* gene and the upstream regulatory mechanism that promotes the proliferation of lake wool papilla cells in vitro. In a previous bioinformatics analysis, we also found that CpG-rich islands existed in the promoter region of the *BMP7* gene, which further indicated that DNA methylation modification may affect the transcriptional expression of the *BMP7* gene promoter by affecting the binding of transcriptional factors [21]. In this study, we found that the mRNA expression level and the core promoter activity of the *BMP7* gene can be increased after DNA demethylation treatment. This result confirms our hypothesis that DNA methylation affects transcription factor binding in the *BMP7* gene promoter region. In addition, it has been reported that DNA methylation plays a regulatory role in hair follicle growth and development by affecting the expression of gene or non-coding RNA [19,38]. We speculate that DNA methylation can affect the growth and development of hair follicles of Hu sheep by acting on the *BMP7* gene. In this study, we first detected the effect of DNA demethylation on the proliferation and cell cycle of Hu sheep DPCs and found that the demethylation inhibitor 5-Aza-dc can promote the proliferation and cell cycle of Hu sheep DPCs. Furthermore, we also detected the effect of DNA methylation on the proliferation and cell cycle of Hu sheep DPCs and found that *DNMT1* can inhibit the proliferation and cell cycle of Hu sheep DPCs. These results further indicate that DNA methylation modification can affect the proliferation and cell cycle of Hu sheep DPCs in vitro by regulating the expression of the *BMP7* gene.

## 5. Conclusions

Our study shows that the differential expression of the BMP7 gene in different patterns of Hu sheep lamb skin may be regulated by DNA methylation modification. In addition, DNA methylation can regulate the proliferation and the percentage of cells in the S phase of DPCs in Hu sheep. These results provide a theoretical basis for the further study of wool bending and the form of the wavy pattern of Hu sheep lamb skin.

## Figures and Tables

**Table 1:** Gene primer sequences used for qRT-PCR.

Gene	Primer Sequence (5′-3′)	Product Size (bp)	Annealing Temperature (°C)	Accession Number
*BMP7*	F: TGAGTTCCGCATTTACAAGG R: GTGGCTGTGATGTCAAAAAC	177	60	NM_001308564.1
*PCNA*	F: CGAGGGCTTCGACACTTAC	97	60	XM_004014340.5
	R: GTCTTCATTGCCAGCACATT			
*DNMT1*	F: CCCAGGAGAAGCAAGTCTGATG R: TGATGGTGGTCTGCCTGGTAGT	91	60	NM_001009473.1
*GAPDH*	F: TCTCAAGGGCATTCTAGGCTAC	151	60	NM_001190390.1
	R: GCCGAATTCATTGTCGTACCAG			

**Table 2:** Primers used for vector construction.

Primer Name	Primer Sequence (5′-3′)	Product Size (bp)	Annealing Temperature (°C)
BMP7 core promoter	F: CGAGCTCTTACGCGTGCTAGCTCCCACGGGTCCCGTTCA R: CAGTACCGGAATGCCAAGCTTCGCGCGACCCGGGCTCCG	800	60
OE-DNMT1	F: TAGTCCAGTGTGGTGGAATTCATGCCTGCCCGTACCGCC	4836	63
	R: GCCCTCTAGACTCGAGCGGCCGCCTAGTCCTTGGCAGCCTCCTC		

## Data Availability

Data are contained within the article.
